# Dynamics of mono‐ and dual‐species biofilm formation and interactions between *Staphylococcus aureus* and Gram‐negative bacteria

**DOI:** 10.1111/1751-7915.12705

**Published:** 2017-04-11

**Authors:** Jitka Makovcova, Vladimir Babak, Pavel Kulich, Josef Masek, Michal Slany, Lenka Cincarova

**Affiliations:** ^1^ Department of Food and Feed SafetyVeterinary Research InstituteBrnoCzech Republic; ^2^ Department of Chemistry and ToxicologyVeterinary Research InstituteBrnoCzech Republic; ^3^ Department of Pharmacology and ImmunotherapyVeterinary Research InstituteBrnoCzech Republic

## Abstract

Microorganisms are not commonly found in the planktonic state but predominantly form dual‐ and multispecies biofilms in almost all natural environments. Bacteria in multispecies biofilms cooperate, compete or have neutral interactions according to the involved species. Here, the development of mono‐ and dual‐species biofilms formed by *Staphylococcus aureus* and other foodborne pathogens such as *Salmonella enterica* subsp. *enterica* serovar *Enteritidis*, potentially pathogenic *Raoultella planticola* and non‐pathogenic *Escherichia coli* over the course of 24, 48 and 72 h was studied. Biofilm formation was evaluated by the crystal violet assay (CV), enumeration of colony‐forming units (CFU cm^−2^) and visualization using confocal laser scanning microscopy (CLSM) and scanning electron microscopy (SEM). In general, Gram‐negative bacterial species and *S. aureus* interacted in a competitive manner. The tested Gram‐negative bacteria grew better in mixed dual‐species biofilms than in their mono‐species biofilms as determined using the CV assay, CFU ml^−2^ enumeration, and CLSM and SEM visualization. In contrast, the growth of *S. aureus* biofilms was reduced when cultured in dual‐species biofilms. CLSM images revealed grape‐like clusters of *S. aureus* and monolayers of Gram‐negative bacteria in both mono‐ and dual‐species biofilms. *S. aureus* clusters in dual‐species biofilms were significantly smaller than clusters in *S. aureus* mono‐species biofilms.

## Introduction

Food‐processing environments provide a variety of conditions, which can favour the formation of biofilms, for example the presence of moisture, nutrients and inocula of microorganisms from the raw materials (Bower *et al*., 1996). Bacterial colonization of food‐processing equipment is a source of damage to metal surfaces (pitting and corrosion) and breakdown of plastics (Mittelman, 1998). Biofilms are defined as microbial communities that are adherent to each other and/or to the surface embedded in self‐produced extracellular polymeric substances (EPS) composed of polysaccharides, proteins, phospholipids, teichoic and even nucleic acids (Costerton *et al*., 1995; Hall‐Stoodley *et al*., 2004; Sauer *et al*., 2007). Microbial cells can adhere to food‐contact surfaces within minutes (Hall‐Stoodley *et al*., 2004), and biofilms can form within hours or days (Schlisselberg and Yaron, 2013). Although the majority of bacteria in the food production environment are non‐pathogenic (Bagge‐Ravn *et al*., 2003; Schirmer *et al*., 2013), these bacteria may be involved in reducing the quality of foods and importantly may facilitate colonization and survival of pathogenic bacteria (Nadell *et al*., 2009; Shi and Zhu, 2009; Van Houdt and Michiels, 2010).

Due to their resistance to disinfectants and sanitizers, biofilms formed by pathogenic microorganisms in food environments can be difficult to completely eliminate from food‐processing facilities. For that reason, procedures for the elimination of biofilms must be optimized, usually on an individual basis for different food‐processing factories. These procedures are based on a combination of physical factors, chemical products and user conditions (Shi and Zhu, 2009; Jahid and Ha, 2012; Srey *et al*., 2013).

The dynamic process of biofilm formation is predominantly characterized by initial reversible attachment of planktonic cells, cell aggregation and colonization of surfaces, biofilm maturation and detachment of cells from the biofilm into a planktonic state (Poulsen, 1999; Costerton *et al*., 2005). Biofilm formation is a general strategy by which microorganisms survive in changing or hostile environments, such as when bacteria are challenged with a limited availability of nutrients, the presence of disinfectants or antibiotics and desiccation or temperature changes (Hall‐Stoodley *et al*., 2004; Bridier *et al*., 2011). Biofilms can be formed by single, dual and/or multiple species of microorganisms and may constitute a single layer or three‐dimensional structures. Mature biofilms represent a highly organized ecosystem with dispersed water channels which ensure the exchange of nutrients, metabolites and waste products (Sauer *et al*., 2007). The close proximity and complex interactions of species within biofilms underlie both synergistic and antagonistic behaviours (Elias and Banin, 2012).

Polymicrobial growth brings with it interspecies interactions that involve communication, typically via quorum sensing, and metabolic cooperation. The interactions within mixed‐species biofilms are suggested to be of a cooperative (synergistic), competitive (antagonistic) or neutral nature based on the genetic background of the involved species (Giaouris *et al*., 2013). Synergistic interactions within biofilms are based on promotion of biofilm formation by co‐aggregation, or metabolic cooperation (one species utilizes a metabolite produced by a neighbouring species), and can also increase resistance to antibiotics or host immune responses compared to mono‐species biofilms. Antagonistic interactions are based on competition over nutrients and growth inhibition (Harriott and Noverr, 2009; Schwering *et al*., 2013). Thus, co‐residence of diverse bacteria in biofilms can lead to an increase or decrease in biomass production (Schwering *et al*., 2013; Ren *et al*., 2014).

*Staphylococcus aureus* and *Salmonella enterica* are two of the most important, globally spread, foodborne pathogens. *Staphylococcus aureus* is a ubiquitous bacterial species commonly found on the skin and hair, as well as in the noses and throats of people and animals. It is the causative agent of a wide spectrum of human infections (Otto, 2013) and is also often responsible for foodborne intoxications through the production of heat‐stable enterotoxins in a variety of food products (Hennekinne *et al*., 2012). *Salmonella enterica* is one of the most significant enteric foodborne bacterial pathogens and is classified into more than 2500 serovars of which the serovars Typhimurium and Enteritidis are the most prevalent. *Salmonella* serovars are responsible for human diseases ranging from gastroenteritis to systemic infections (Ruby *et al*., 2012; Foley *et al*., 2013). *Escherichia coli* is primarily a commensal species which constitutes part of the physiological microflora of the colon and distal ileum. However, *E. coli* also includes important foodborne pathogenic strains causing intestinal or extraintestinal infections (Kaper *et al*., 2004). *Raoultella planticola* (formerly *Klebsiella*) is generally considered to be an environmental bacterium found in soil and water (Drancourt *et al*., 2001) and rarely causes clinical infections. However, this organism was the reported pathogen in several cases of serious infection (Olson *et al*., 2012; Koukoulaki *et al*., 2014). *R. planticola* is an important histamine‐producing bacterium in fish, which causes foodborne intoxication due to histamine fish poisoning (Taylor, 1986; Lehane and Olley, 2000).

The above‐mentioned bacterial genera and species are able to form biofilms on different surfaces commonly used in the food industry such as glass, plastic or metal (O'Toole *et al*., 2000; Oliveira *et al*., 2007). Besides stainless steel, plastic materials are still frequently used in the food industry for the construction of tanks, pipeworks, accessories and cutting surfaces (Pompermayer and Gaylarde, 2000). Various methods, both culture dependent and culture independent, have been developed to study the structure of multispecies biofilms and interactions between different species in various foods and food‐contact surfaces (Giaouris *et al*., 2013; Schwering *et al*., 2013).

Although various studies have highlighted the importance of multispecies biofilms in foods and food environments, research on this topic is still in an early phase (Manuzon and Blaschek, 2007; Moons *et al*., 2009). The vast majority of studies have focused on either mixed Gram‐negative or Gram‐positive biofilms or mixed biofilms consisting of bacteria and fungi. There are only a few detailed studies devoted to dual‐species biofilms composed of Gram‐positive bacteria, specifically *S. aureus* and Gram‐negative bacteria (Peters *et al*., 2010; Giaouris *et al*., 2015). Hence, the aim of our work was to study mono‐ and dual‐species biofilms of *S. aureus* and three different Gram‐negative bacteria, *S. enterica*,* E. coli* and *R. planticola*, and to evaluate interactions between them over the course of 3 days using both quantitative assays (CFU, total biomass) and qualitative methods, namely confocal laser scanning microscopy (CLSM) and Scanning electron microscopy (SEM).

## Results

### Total biomass quantification

**Figure 1:**
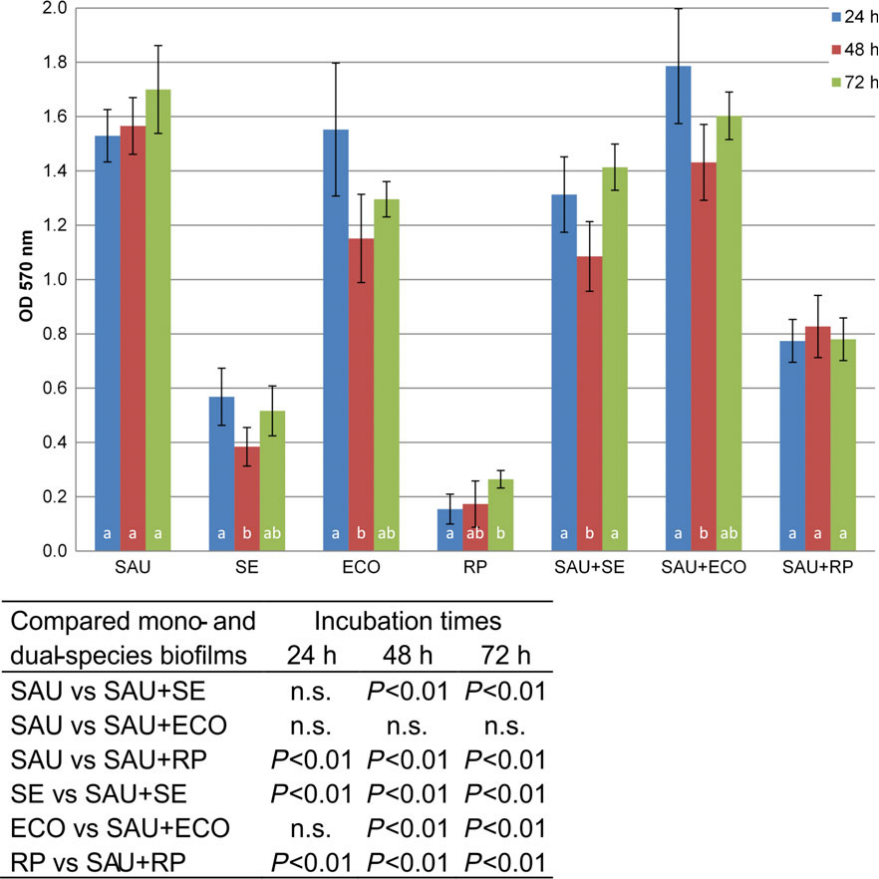
Crystal violet quantification of mono‐ and dual‐species biofilms of *Staphylococcus aureus* and Gram‐negative bacteria. The columns represent mean values of OD 570 nm; the vertical bars denote the 95% confidence intervals of these means. Letters above the *x*‐axis denote statistically significant differences (*P* < 0.05 at least) among incubation times (columns sharing the same letter are not significantly different from each other; columns that have no letter in common are significantly different from each other). The table embedded within the figure shows the significance of differences in OD 570 nm between mono‐ and dual‐species biofilms (ANOVA followed by *post hoc* Bonferroni multiple comparison tests).

The total biomass of monoculture biofilms was compared to dual‐species biofilms formed by *S. aureus* in the presence of Gram‐negative bacteria after 24, 48 and 72 h of incubation at 25 °C (Fig. 1). The biofilm formation capacity of the tested *S. aureus* and Gram‐negative bacteria was determined using the criteria of Śtepanović and colleagues (Malone *et al*., 2009), in TSB at 25 °C. *S. aureus* and *E. coli* strains used in this study are moderate biofilm formers, while *S. enterica* strain is a weak biofilm former and the *R. planticola* strain forms biofilm so weakly that according to the Śtepanović criteria, it should be classified as an isolate that does not form biofilm at all. Mean OD 570 nm values of dual‐species biofilms formed by *S. aureus* and Gram‐negative bacteria were statistically significantly higher, compared to mono‐species biofilms of Gram‐negative bacteria in all three incubation periods (*P* < 0.01; ANOVA, Bonferroni tests), except for *S. aureus* co‐cultured with *E. coli* compared to mono‐species biofilms after the 24 h incubation time (*P* > 0.05). In contrast, when *S. aureus* was co‐cultured with *S. enterica* or *R. planticola*, mean OD 570 nm values were statistically significantly lower compared to monoculture of *S. aureus* biofilms in all incubation times, except for a higher mean OD 570 nm value for co‐culture of *S. aureus* with *S. enterica* after 24 h of incubation (*P* > 0.05).

### Quantification of viable cells in biofilms

**Figure 2:**
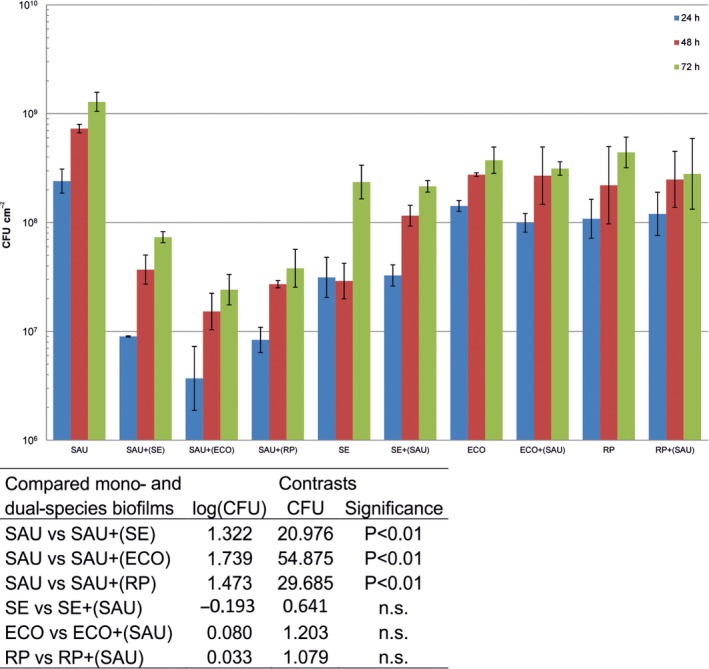
Viable cell counts of mono‐ and dual‐species biofilms. The columns represent values of geometric means of CFU cm^−2^; vertical bars correspond to geometric standard deviations. The *y*‐axis is scaled logarithmically. Table 1b shows the significance of differences between mono‐ and dual‐species biofilms (ANCOVA followed by a testing of contrasts). The slope of the covariate (incubation time) is 0.014, that is CFU cm^−2^ increased on average 2.16× (*P* < 0.01) in 24 h. SAU,* S. aureus*; SE,* S. enterica*; ECO,* E. coli*; RP,* R. planticola*; SAU+SE, co‐culture of *S. aureus* and *S. enterica*, etc.; SAU+(SE), number of viable *S. aureus* cells when co‐cultured with *S. enterica*; SE+(SAU). Number of viable *S. enterica* cells when co‐cultured with *S. aureus*, etc.; n.s. non‐significant.

Colony‐forming unit numbers of *S. aureus* and Gram‐negative bacterial strains in single‐ and dual‐species biofilms were enumerated using a plating method after 24, 48 and 72 h of incubation at 25 °C (Fig. 2). *S. aureus*,* S. enterica*,* E. coli* and *R. planticola* were able to adhere and to form biofilms both in single as well as in mixed cultures. The number of attached cells of *S. aureus*,* S. enterica*,* E. coli* or *R. planticola* on the polystyrene surface over the time of incubation assayed ranged between 2.40 × 10^8^ and 1.28 × 10^9^ for *S. aureus*, 3.13 × 10^7^ and 2.36 × 10^8^ for *S. enterica*, 1.42 × 10^8^ and 3.74 × 10^8^ for *E. coli* and 1.08 × 10^8^ and 4.41 × 10^8^ CFU cm^−2^ for *R. planticola* respectively. Fig. 2 shows that the CFU cm^−2^ values of all four microorganisms increased with the length of incubation; the slope of covariate (incubation time) was 0.014; that is, CFU cm^−2^ increased on average 2.16× (*P* < 0.01; ANCOVA) in 24 h. Cell numbers of all tested Gram‐negative bacteria were not significantly affected when co‐cultured with *S. aureus* compared to mono‐species biofilms (*P* > 0.05; ANCOVA, contrasts). The data showed that co‐culture of *S. aureus* with Gram‐negative bacteria in dual‐species biofilms resulted in a significant reduction in the counts of *S. aureus* (*P* < 0.01; ANCOVA, contrasts). The reduction in the counts of *S. aureus* with *S. enterica* was 21x, with *E. coli* 55× and with *R. planticola* 30× respectively (Table in Fig. 2).

### Confocal laser scanning microscopy

**Figure 3:**
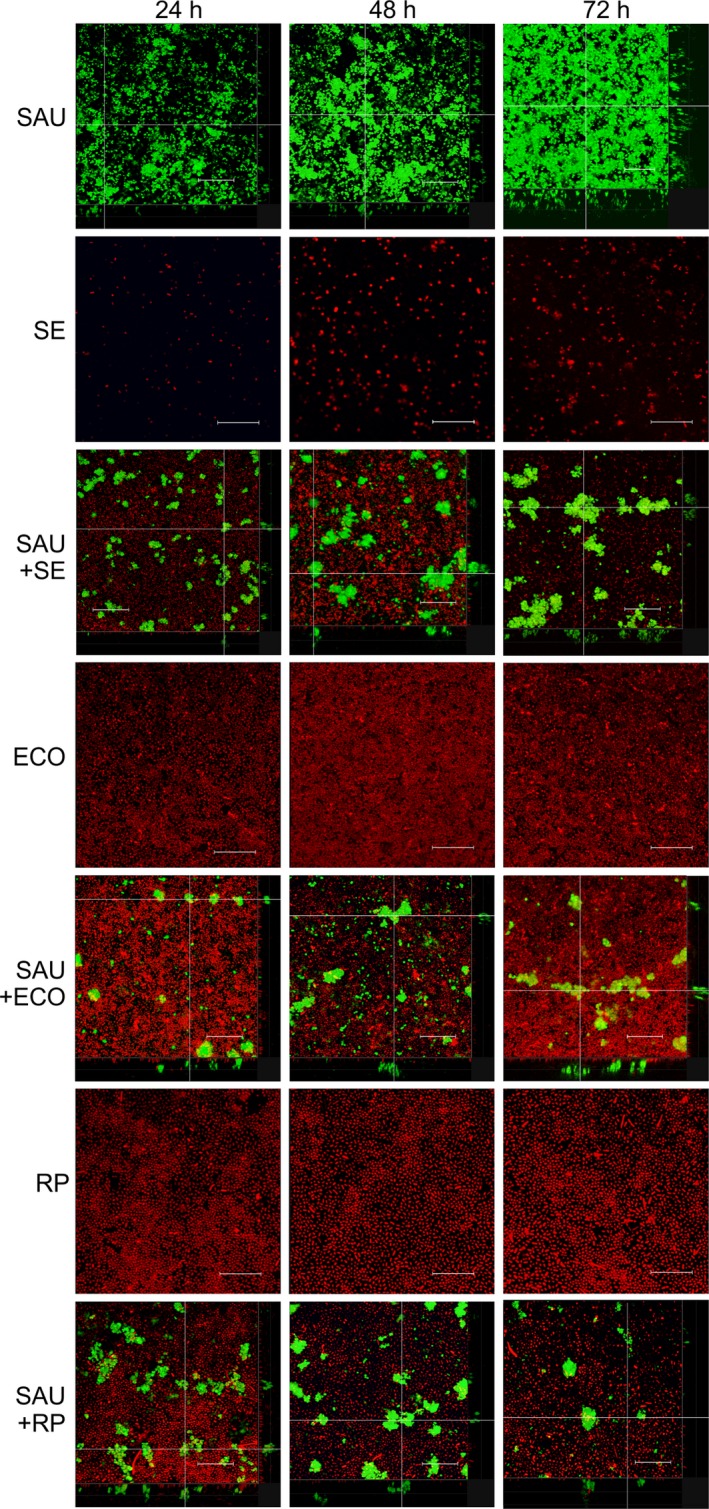
Representative CLSM images of monocultures and dual‐species biofilms formed by *Staphylococcus aureus* and Gram‐negative bacteria. SAU,* S. aureus*; SE,* Salmonella enterica*; ECO,* E. coli*; RP,* R. planticola*; SAU+SE,* S. aureus* and *S. enterica* dual‐species biofilms, etc. Scale bar represents 25 μm.

To confirm the results obtained with the CFU and crystal violet assay, biofilms were visualized using CLSM. Mono‐species biofilms of *S. aureus*,* S. enterica*,* E. coli* and *R. planticola* and the influence of Gram‐negative bacteria on the development of *S. aureus* cells in mixed‐species biofilms were investigated after 24, 48 and 72 h at 25 C. Because different architectures of *S. aureus* mono‐species biofilms and dual‐species biofilms with Gram‐negative bacteria in individual rows were observed, all *z*‐stacks were composed by the transparent snapshot with the cross section and side panels in the positions indicated by the dashed lines. While the majority of fluorescent emission overlap between GFP and mCherry was eliminated by the appropriate setting of the emission filters, a small amount of fluorescent overlap was recorded as yellow colour. Figure 3 shows representative CLSM micrographs of both mono‐ and dual‐species biofilm development after 24, 48 and 72 h.

Confocal laser scanning microscopy confirmed, in general, that Gram‐negative bacteria had a suppressive effect on *S. aureus* at every stage of biofilm formation, in comparison with the mono‐species counterpart. Gram‐negative bacteria, both in mono‐species biofilms or in mixed cultures with *S. aureus*, formed monolayers over time. Individual cells of *S. enterica* were evident after 24 h, and after 48 and 72 h of culture, sparsely developed biofilms were visible. Mono‐species biofilms of *E. coli* and *R. planticola* were much denser already after 24 h compared to *S. enterica* biofilms. Monolayers were relatively homogenous, with occasional small holes; sometimes small clusters of bacteria were evident. In contrast, *S. aureus* formed three‐dimensional structures, and formed biofilms were dense and included holes of different sizes and channels which thickened with the duration of incubation. In the dual‐species biofilms, Gram‐negative cells formed patchy monolayers on the bottom of the wells. Cells were seen to be more evenly attached especially around *S. aureus* clusters, or some of them were found on the surface of grape‐like clusters of *S. aureus* (see side panels) and then protruded into the space during incubation. When *S. aureus* was co‐cultured with Gram‐negative bacteria, clusters were not as robust as compared to mono‐species biofilms. The images show that Gram‐negative bacteria were attached to the bottom of the well and that *S. aureus* cells attached and formed grape‐like structures on Gram‐negative bacterial monolayers. The thickness of *S. aureus* biofilms increased both in monocultures and when co‐cultured with Gram‐negative bacteria over 24, 48 and 72 h of incubation. The thickness of *S. aureus* biofilms was approximately 15, 20 and 27 μm. In dual‐species biofilms, *S. aureus* clusters were thinner than their corresponding mono‐species variants. When *S. aureus* was co‐cultured with *S. enterica*, the thickness of biofilms was 15 and 20 μm after 24 and 48 h, but then fell to 15 μm after 72 h. In dual‐species biofilms formed with *E. coli*,* S. aureus* formed biofilms with thicknesses of 10, 13 and 13 μm. *S. aureus* clusters were 10, 15 and 15 μm thick, when co‐cultured with *R. planticola*. Gram‐negative bacteria formed mono‐layered biofilms throughout the incubation time both in mono‐ and dual‐species biofilms. The depth of these slices was not measured as they were smaller than 5 μm.

Confocal laser scanning microscopy images showed that the dual‐species biofilms were not composed of both species mixed together in a typical co‐aggregation structure but were rather characterized by separate spatial clusters of *S. aureus* and monolayers of Gram‐negative bacteria. This confirmed our previous results, where we observed *S. aureus* to have high aggregation propensity (80% aggregation after 24 h) and Gram‐negative bacteria to possess moderate aggregation propensity (40–60% aggregation after 24 h; data not shown). However, in mixed culture, the co‐aggregation was very low for *S. aureus* and totally absent in Gram‐negative bacteria.

### Scanning electron microscopy

**Figure 4:**
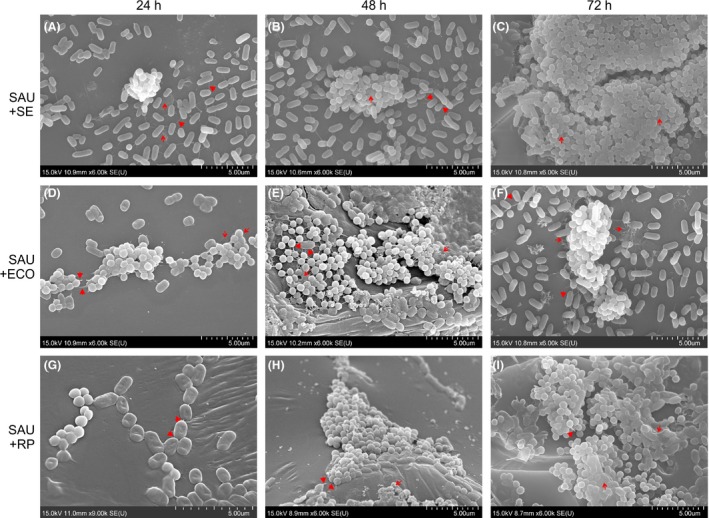
Representative scanning electron microscopy images of dual‐species biofilms formed by *Staphylococcus aureus* and Gram‐negative bacteria. SAU+SE *S. aureus* and *Salmonella enterica*, SAU+ECO *S. aureus* and *E. coli*, SAU+RP *S. aureus* and *R. planticola* dual‐species biofilms. Arrows: amorphous extracellular matrix; tips: adhesive fibres.

Scanning electron microscopy was used to examine the structure and interactions in static biofilms formed by *S. aureus* and Gram‐negative bacteria after 24, 48 and 72 h of incubation at 25 °C. SEM showed that mixed biofilms consisted of *S. aureus* microcolonies scattered across the surface and Gram‐negative bacteria attached to the surface in monolayers. Over the course of co‐culture, *S. aureus* grape‐like clusters gained in volume, while the number of Gram‐negative bacterial cells increased, but remained in monolayers (Fig. 4).

**Figure 5:**
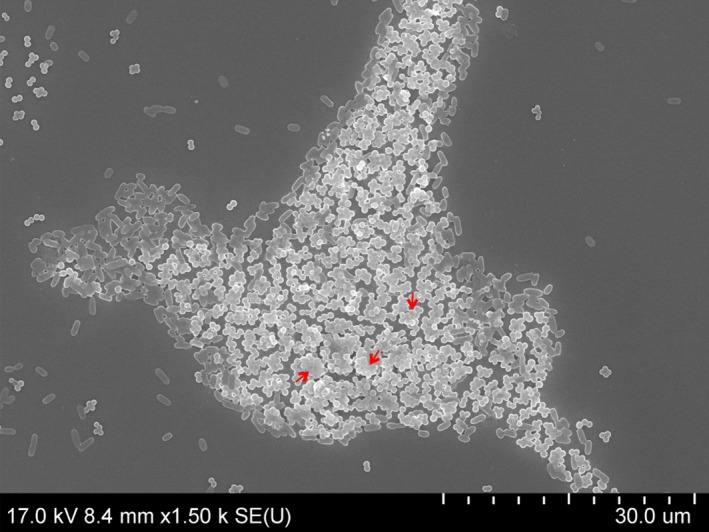
Scanning electron microscopy image of a *Staphylococcus aureus* microcolony surrounded by *Salmonella enterica* cells, dual‐species biofilm after 72 h. Arrows: amorphous extracellular matrix.

There was evidence that Gram‐negative bacteria surrounded *S. aureus* clusters and that some of them had been incorporated into clusters or had attached to the top of them. Cells could be seen that were embedded in an amorphous matrix (arrow; Fig. 5).

A close‐up, side view image of 24 h dual‐species biofilms showed an attached *S. enterica* cell on the surface, to which *S. aureus* cell clusters clung. Amorphous matrix, possibly EPS (arrow), was evident on the surface of cocci.

**Figure 6:**
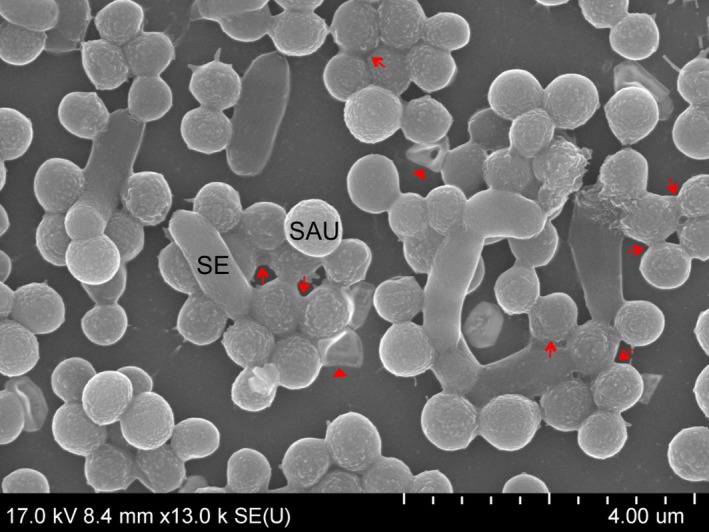
Scanning electron microscopy close‐up image of *Staphylococcus aureus* and *Salmonella enterica* dual‐species biofilm after 72 h. Arrows: amorphous extracellular matrix; tips: adhesive fibres.

Nascent and fully formed cell‐to‐cell connections, Gram‐negative cells attached to the surface and to other cells using fibril‐like structures (tip) and intercellular slime connecting the biofilms (arrow) were seen after 24 h (Fig. 4A, D and G). A few putative outer membrane vesicles (OMVs) were observed on the surfaces of *E. coli* and *R. planticola* cells. Most cells had a normal shape and a smooth cell surface. After 48 h of incubation, the number of fibril‐like junctures and cell‐to‐cell connections between cells had multiplied (tip) and amorphous mass (arrow) had increased (Fig. 4B and E). Buds and putative membrane vesicles (MVs) formed by *S. aureus* and putative OMVs on the surface of *E. coli* and *R. planticolla* cells were evident (Fig. 4E and H). Damaged cells showed a rough and shrunken appearance, and deformation or cellular debris was evident, especially in co‐culture of *S. aureus* and *E. coli* (Fig. 4E). Images of 72 h co‐culture of *S. aureus* and *S. enterica* and *S. aureus* and *R. planticola* showed biofilms embedded in a large amount of possible EPS (arrow), (Fig. 4C and I). A close‐up image of 72 h biofilm formed by *S. aureus* and *S. enterica* revealed an apparent EPS matrix surrounding cells. Damaged cells of *S. aureus* were rough with individual bumps and buds and were deformed on their surfaces. Also, some Gram‐negative cells showed signs of damage. Detailed images revealed the first cell‐to‐cell connections and EPS, but also lysed cells and cell debris (Fig. 6).

## Discussion

Biofilms are found in different environments and are usually not formed by single species, but rather by dual or multiple species. Cell‐to‐cell interactions influence the temporal and spatial organization of biofilm architecture and can be categorized as either cooperative or competitive (Elias and Banin, 2012; Rendueles and Ghigo, 2012).

Attention has been paid to bacterial foodborne pathogens and intra‐ and interspecies interactions between Gram‐positive *Staphylococcus aureus* and the Gram‐negative bacteria *Salmonella enterica, Raoultella planticola,* or *Escherichia coli*. Because S*. aureus* and *S. enterica* are known to be important foodborne pathogens, we considered it important to study their interspecies interactions. Furthermore, we considered it interesting to include potentially pathogenic *R. planticola* and non‐pathogenic *E. coli*, both obtained from food‐processing environments after sanitation, in our study.

The results measured in the crystal violet assay showed that *S. aureus*,* S. enterica* and *E. coli* were able to form mono‐species biofilms after 24 h at 25 °C to varying degrees, while *R. planticola* is not a biofilm former. However, *R. planticola* is a species that produces capsules (Shaikh*,* 2011). Capsules generally are stained very poorly with reagents used in simple crystal violet staining (Breakwell *et al*., 2009) and thus can be a source of inaccuracy – the total biofilm biomass could be underestimated when simple crystal violet staining is used for determination. Total biomass volume was decreased in dual‐species biofilms (except for co‐culture of *S. aureus* and *E. coli* after 24 h) compared to *S. aureus* mono‐species biofilms over time. These results indicated a competitive relationship between bacteria, an idea which is supported by the obtained CFU data. In mono‐ and dual‐species biofilms, there was a gradual increase in CFU values over time. In dual‐species biofilms, the CFU values of Gram‐negative bacteria remained almost unchanged, while *S. aureus* CFU values significantly decreased compared to single biofilms of the same species (Fig. 2). This clearly indicates an inhibitory effect of Gram‐negative bacteria on *S. aureus* cells and suggests that their overall interactions are competitive rather than cooperative.

There are several studies regarding formation of dual biofilms by *S. aureus* and Gram‐negative bacteria. A competitive relationship was observed by Millezi *et al*., 2012; between *S. aureus* and *E. coli*, in which the number of viable *S. aureus* cells in biofilms was diminished by the presence of *E. coli* (Millezi *et al*., 2012). Likewise, Pompermayer and Gaylarde, 2000; investigated the adherence of *S. aureus* and *E. coli* and concluded that there is a competition between bacteria with *E. coli* being favoured in dual‐species culture (Pompermayer and Gaylarde, 2000). The authors suggested that the adherence of *E. coli* could be greater because of the shorter generation time of this bacterium, which could enable it to develop and maintain dominance. Similarly, this could be the reason for the inhibition of *S. aureus* in our case, because all the Gram‐negative bacteria used in our experiments have shorter generation times than *S. aureus*.

It is well recognized that the exopolysaccharide capsule is one of the key bacterial components for biofilm formation (Wang *et al*., 2015). Capsules are produced by many microorganisms, including *Escherichia*,* Salmonella, Klebsiella or Staphylococcus* strains, and can be either adhesive or anti‐adhesive (Hassan and Frank, 2004; Coldren, 2009). Valle *et al*. (2006) demonstrated that *E. coli* expressing group II capsules released a soluble polysaccharide that induces physiochemical surface alterations, which prevent adhesion and biofilm formation by a wide range of both Gram‐negative and Gram‐positive bacteria, including *S. aureus*. Similarly, an *E. coli* biofilm‐associated anti‐adhesion polysaccharide which reduces susceptibility to invasion and resulted in rapid exclusion of *S. aureus* from mixed *E. coli* and *S. aureus* biofilms was identified (Rendueles *et al*., 2011).

Only a few publications have focused on *S. aureus* and *S. enterica* dual‐species biofilm formation. In dual‐species biofilms formed by *S. aureus* and *S. enterica* serovar Typhimurium in a fermentor, *S. aureus* dominated (99%) over *S*. Typhimurium (Knowles *et al*., 2005). Given that this experiment is vastly different from the ones described here, the results are not comparable. The development of dual‐species biofilms formed by *S. aureus* and *S. enterica* serotype Enteritidis was described by Zhang *et al*. (2014). Mixed biofilms were quantified using colony‐forming units and crystal violet assays and *S. aureus* predominated. Biofilm formation was performed at 37 °C, in contrast to our study, in which experiments were performed at 25 °C. It was observed in other studies that biofilm formation of *S. aureus* as well as other species differs depending on the cultivation temperature (Hoštacká *et al*., 2010; de Souza *et al*., 2014; Pavlovsky *et al*., 2015). Moreover, mono‐species biofilms of individual species were not evaluated. Therefore, the relationship between bacteria cannot be assessed. Blana and co‐authors (Blana *et al*., 2015) found that *luxS*‐positive *Salmonella enterica* serotype Typhimurium culture supernatant significantly increased the *S. aureus* single‐cell lag time. We may conclude that the extended lag time of *S. aureus* gives an advantage to *Salmonella* in the covering of surfaces and the formation of biofilms. Unfortunately, to our knowledge, there has been no study regarding dual‐species biofilm formation by *S. aureus* and *R. planticola* (previously *K. pneumoniae*).

Other microbes have also been described to show altered characteristics due to interspecies interactions and to exhibit different properties depending on whether they grow in mono‐ or dual‐species biofilms. Varposhti *et al*., 2014; observed cooperation between *Pseudomonas aeruginosa* and *Acinetobacter baumannii* and *Stenotrophomonas maltophilia*, pathogenic bacteria from the respiratory tract, when cultivated in dual‐species biofilms (Varposhti *et al*., 2014). Giaouris *et al*., 2013; also observed beneficial cooperation: co‐culture with *Listeria monocytogenes* within a dual‐species biofilm community strongly increased the resistance of *Pseudomonas putida* to benzalkonium chloride (Giaouris *et al*., 2013). Peters *et al*., 2010; in contrast, observed cooperation between *S. aureus* and the fungal species *Candida albicans* based on physical interactions and differential regulation of specific virulence factors (Peters *et al*., 2010). They observed an enhanced pathogenesis of *S. aureus* mediated by the association of *S. aureus* with hyphal elements of *Candida albicans* that can penetrate through epithelial layers. Peters *et al*. also observed enhanced virulence of *S. aureus* during co‐infection with *Candida albicans*: differential protein expression analysis revealed downregulation of the global transcriptional repressor of virulence factors, CodY, and, as a consequence of this, upregulated expression of *S. aureus* virulence factors.

The competition of microbes can be manifested by the changing of their local environment either directly or as a consequence of their secondary metabolism and physiological by‐products. For example, *Lactobacilli* spp. produce lactic acid that lowers environmental pH and thus limits the growth of other species such as *Neisseria gonorrhoeae* (Graver and Wade, 2011). Bacteria also use low‐molecular weight compounds (toxic metabolic by‐products, bacteriocins or colicins). *Streptococcus pneumoniae* produces hydrogen peroxide as a toxic by‐product of aerobic metabolism. In the human nasopharynx, it is an inhibitor of *Neisseria meningitidis* and *Moraxella catarrhalis* (Pericone *et al*., 2000). Other mechanisms of competition in multispecies biofilms including contact‐dependent growth inhibition and predation were characterized by Rendueles *et al*. (2012).

Confocal laser scanning microscopy and SEM were used to study the complexity and structural heterogeneity of mono‐ and dual‐species biofilms. CLSM is a widely used tool for the observation of biofilms because it allows one to obtain a three‐dimensional image of the structure of a biofilm and to monitor its development over time without harmful effects on its growth (Canette and Briandet, 2014). CLSM images (Fig. 3) were in agreement with the CFU data (Fig. 2): whereas Gram‐negative bacteria are not negatively affected by the presence of *S. aureus*,* S. aureu*s is inhibited when co‐cultured with Gram‐negative bacteria. In CLSM scanning, the projection of captured images on the *z*‐axis (data not shown) showed that Gram‐negative bacteria were attached to the bottom of the well and that *S. aureus* cells adhered and formed three‐dimensional, grape‐like structures on Gram‐negative bacterial monolayers. CLSM images showed that the dual‐species biofilms were not composed of both species mixed together in a typical co‐aggregation structure that is typical for mixed biofilms composed of species cooperating or interacting synergistically (Elias and Banin, 2012). Rather, in this case, the observed inability of the species to co‐aggregate together with the total biomass data and CFU (Figs 1 and 2) show that *S. aureus* and Gram‐negative bacteria compete in mixed‐species biofilms.

Using CLSM, different biofilm architectures for mono‐ and dual‐species biofilms composed by *Staphylococcus piscifermentans* and *Salmonella* Agona were observed (Habimana *et al*., 2010). Habimana *et al*., 2010, described biofilms composed by *Staphylococcus piscifermentans* and *Salmonella* Agona. While *Staphylococcus* mono‐species biofilms were defined as compact, with the presence of holes in the matrix, *S*. *Agona* mono‐species biofilms were found to be composed of more channels. In mixed‐species biofilms, *S. Agona* cells were found to partially cover *Staphylococcus* microcolony niches. This architecture is similar to our results, but in our study, the biofilms formed by the *Salmonella* strain were not so dense and compact and *Staphylococcus* did not promote *Salmonella* biofilm formation in mixed‐species biofilms. Unfortunately, we could not find any publications that would describe dual‐species biofilm formation specifically by *S. aureus* and the Gram‐negative bacterial species used in our study using CLSM.

Data obtained using CLSM, CV staining and CFU enumeration were all in conformity with each other, with some exceptions. Differences could arise due to the different principles of these methods. CV staining is a more robust method used for quantification of total biomass (simple CV staining labels living and dead cells as well as the extracellular matrix). In our set‐up, CLSM visualizes only cells containing the GFP protein regardless of whether the cells are dead or alive or VBNC (Viable but non‐culturable). CLSM does not quantify the extracellular matrix. CV quantifies total biofilm biomass. CFU, in contrast, is a measurement that quantifies changes only in cells that are viable and culturable, and the accuracy heavily depends on the preparation of the sample. CLSM‐based visualization of *S. aureus* clearly showed an increase in density and thickness over 24, 48 and 72 h, whereas the CV and CFU data revealed no pronounced increase in total biofilm biomass or in viable and culturable cells at the measured time points. One reason for this inaccuracy may lie in the fact that CFU quantifies only viable and culturable cells; however, a certain proportion of the cells in the biofilms are dead (Bayles, 2007) or non‐cultivatable cells, and these are quantified by CLSM but not by CFU enumeration. For CLSM, it is necessary to use special culture plates, which differ slightly in cultivation area and material. This can affect the resulting biofilms that are formed, and therefore, it may not be accurate to compare individual results of different methods.

While CLSM revealed a significant increase in the total cell number of *S. aureus* at 24 h versus 48 h versus 72 h, CV staining showed a relatively unimportant increase in total biofilm quantity. The most significant increase in biofilm biomass would probably occur in the time interval 0–24 h. In this interval, the bacterial cells of the tested isolates mainly adhere to the surface, produce extracellular matrix and multiply. After approximately 24 h, cells that are already part of the biofilm tend to form biofilm mass and multiply less within the biofilm. Thus, the overall biofilm biomass no longer substantially changes in this phase (this is strongly dependent on conditions – e.g. presence of stress factor).

In the case of *S. enterica*, CLSM and CFU data corresponded with each other. The changes in biofilm quantity revealed in the CV data were not statistically significant for all time points. This could be due to the fact that the tested *Salmonella enterica* isolate is a weak biofilm former and thus is less stable during CV staining. For *Escherichia coli*, CV quantification also did not reveal any significant changes and CFU and CLSM data were in agreement with this result. According to the classification of Stepanović *et al*., 2004; *Raoultella planticolla* forms biofilm only very weakly. CV data showed no significant increase in quantity between 24 and 72 h. Enumeration of CFU revealed a significant increase in the number of viable cells between 24 and 72 h, an observation that was confirmed using CLSM.

**Figure 7:**
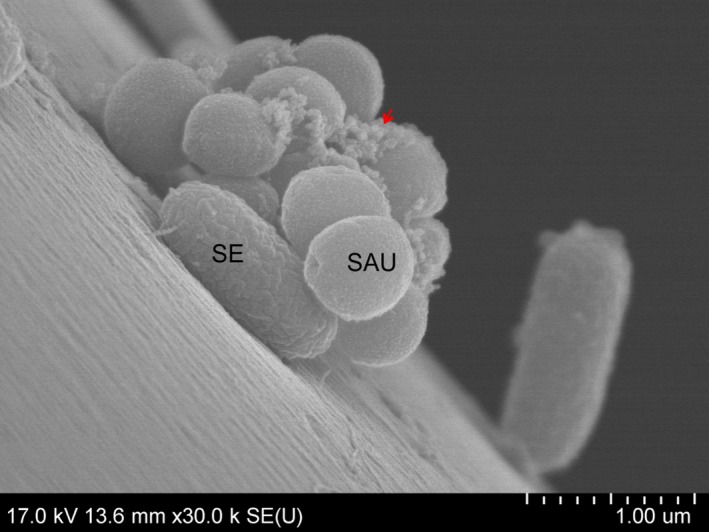
Scanning electron microscopy detail image of adhesion of *Staphylococcus aureus* and *Salmonella enterica* after 24 h. Arrows: amorphous extracellular matrix.

Scanning electron microscopy permits visualization of detailed surface morphologies of microbial biofilms and their structures. SEM images confirmed the CLSM analysis, where Gram‐negative bacteria formed monolayers over the surface and *S. aureus* created grape‐like structures adhering to Gram‐negative bacteria. With increasing incubation times, the number of bacterial cells increased, but damaged or dead cells also appeared. With increasing duration of incubation time, nascent and then established cell‐to‐cell connections and an increasing amount of amorphous matrix, probably EPS, were clearly visible. In the case of *S. enterica*, roughened cell surfaces, cellular deformation and the formation of depressions in some *S. aureus* cocci were indicative of cellular damage (Fig. 7). Putative membrane vesicles formed by *S. aureus* and *E. coli* or *R. planticola* outer membrane vesicles were observed. A variety of pathogenic Gram‐negative and environmental bacteria secrete OMVs during growth. The production of OMVs has among other functions, a role in cell‐to‐cell communication (Kulp and Kuehn, 2010). OMVs associated with biofilm production have been studied most extensively for *P. aeruginosa*, where they comprise a half of the total lipopolysaccharide content and are associated with the entire biofilm matrix (Schooling and Beveridge, 2006); they have also been described to stimulate biofilm production in *Helicobacter pylori* (Yonezawa *et al*., 2009). This result suggests that vesicles enable biofilms to form and that their presence in biofilms is not solely the result of their entrapment in the matrix. However, little is known about the MVs produced by Gram‐positive bacteria. Lee *et al*. (2009) first demonstrated that *S. aureus* release MVs to the extracellular environment during *in vitro* culture and virulence‐associated proteins were identified in *S. aureus* MVs *in vivo* and *in vitro* (Gurung *et al*., 2011; Thay *et al*., 2013). Unfortunately, again, we found no studies of dual‐species biofilms formed by *S. aureus* and *S. enterica*,* E. coli* or *R. planticola* supported by SEM analysis.

We believe that a better understanding of the interactions in dual‐ and multispecies biofilms formed by both foodborne pathogenic and non‐pathogenic bacteria occurring in the food industry can lead to important new insights that will facilitate the control of biofilm formation in food‐processing environments and thus to an improvement in food safety.

## Experimental procedures

### Bacterial strains and growth conditions

In this study, *Staphylococcus aureus* subsp. *aureus* RN4220 (Novick, 1990), a kind gift from Julien Deschamps, French National Institute for Agriculture Research, was used. *Salmonella enterica* subsp. *enterica* serovar Enteritidis 147 originated from egg content (Methner *et al*., 1995); *Escherichia coli* 1685 was isolated from contact surfaces in a meat‐processing plant, and *Raoultella planticola* 191 was isolated from contact surfaces in a dairy. Samples originating from food‐contact surfaces from meat and dairy‐processing plants were collected within 2 h after sanitation by swabbing. Swabs were washed in PBS and subsequently plated onto MacConkey agar. Single colonies were subcultured and identified using ENTEROtest 24 (Erba Lachema s. r. o., Brno, Czech Republic). The identity of all strains was proven by sequencing analysis of two different loci of the *16S rRNA* gene (Harmsen *et al*., 2003; Slany *et al*., 2007). Blastn comparison with a publicly available database (http://www.ezbiocloud.net) revealed homology of higher than 99.5% (all analysed strains). The strains were stored in trypticase soy broth (TSB; Oxoid, England) supplemented with 25% glycerol (Lach‐ner, s. r. o, Neratovice, Czech Republic) at −80 °C. For strain reactivation and use, an aliquot of freezing culture medium was subcultured on trypticase soy agar (TSA; Oxoid., Hampshire, England) and incubated at 37 °C for 24 h. A single colony from a plate was inoculated into 5 ml of TSB and incubated statically for 17 h at 37 °C.

### Preparation of mono‐ and dual‐species cultures

Bacterial strains were grown in TSB at 37 °C to the exponential phase of growth (approximately 10^9^ CFU ml^−1^). Bacterial cells were then pelleted at 4000× *g* for 20 min, and the cell pellet was resuspended in 5 ml of fresh TSB. Optical densities (ODs) of bacterial suspensions were measured using a spectrophotometer (BioPhotometer, Eppendorf, Hamburg, Germany) and adjusted to an absorbance of 1.0 at 600 nm (approximately 10^8^ CFU ml^−1^). Individual bacterial strains were diluted 1:100 in fresh TSB. An equal volume of the two 1:100 diluted mono‐species cultures was combined to make the dual‐species culture. The cell numbers of individual strains were confirmed using a direct plating method on TSA plates (the final amount of diluted culture was 5 × 10^6^).

### Total biomass quantification

Formation of mono‐ and dual‐species biofilms was quantified using the crystal violet (CV) staining method (Stepanović *et al*., 2007) with slight modifications. Mono‐ and dual‐species cultures, 1000 μl per well, were aseptically dispensed in six replicates to sterile polystyrene, flat‐bottom 48‐well tissue culture plates (Jet Biofil Jet Bio‐Filtration, Guangzhou, China). As a negative control, 1000 μl of TSB only was used. Plates were incubated statically for 24, 48 and 72 h at 25 °C. The culture medium was refreshed every 24 h. After the incubation period, planktonic cells were aspirated carefully and the wells were washed three times with phosphate‐buffered saline (PBS; pH 7.2). The plates were inverted and allowed to air‐dry at room temperature. The remaining attached bacteria were fixed with 1000 μl of 99% methanol per well, and after 15 min, plates were emptied and air‐dried. Subsequently, the biofilms were stained using 1000 μl of 0.25% crystal violet solution per well, followed by 20 min incubation at room temperature. Excess unbound dye was removed by thoroughly washing the plates with distilled water. Finally, after the plates were air‐dried, stained biofilms were resolubilized with 750 μl of an ethanol:acetone mixture (80:20) per well and incubated for 20 min at room temperature. The OD of each well was measured at 570 nm, using a microtitre plate reader (spectrophotometer Synergy H1 HYBRID Reader; BioTek, Swindon, UK), and biofilm mass was expressed as OD 570 nm values. The assay was repeated three times.

### Quantification of viable cells in biofilm

The quantification of viable cells in single‐ or dual‐species biofilms was determined in four‐well polystyrene‐bottomed plates with a microwell size of 15 mm (In Vitro Scientific, USA) using the plate counting technique. For removal of cells from the bottom of the well, sterile pipette tip scraping and repeated pipetting of adhered cells was used. The number of viable cells was determined using TSA in mono‐species biofilms. In dual‐species biofilms, MacConkey agar for *S. enterica*,* E. coli* and *R. planticola* and Columbia blood agar with colistin sulfate (10 mg l^−1^) and nalidixic acid (10 mg l^−1^) for *S. aureus* were used. The dishes were incubated at 37 °C for 24 h. The values were expressed as CFU cm^−2^. Assays were performed in biological and technical triplicates.

### Confocal laser scanning microscopy

To observe the development of mono‐species biofilms and interactions of bacteria in dual‐species biofilms, fluorescent protein‐labelled bacteria were constructed. Briefly, the *S. aureus* subsp. *aureus* RN4220 (a kind gift from Julien Deschamps, French National Institute for Agriculture Research) was transformated by expression plasmid pFPV25.1 [gift from Raphael Valdivia (Valdivia and Falkow, 1996); Addgene plasmid # 20668] producing a reporter protein GFP. Tested Gram‐negative bacteria were prepared by cloning of a mCherry coding sequence from pLV‐mCherry [gift from Pantelis Tsoulfas; Addgene plasmid # 36084, (Dull *et al*., 1998)] into plasmid pFPV25.1. This plasmid was transformed into Gram‐negative bacteria according to a previously published procedure (Gonzales *et al*., 2013). Commercially available sterilized μ‐slide eight‐well ibiTreat coverslips (IBIDI, Martinsried, Germany) were used to prepare mono‐ and dual‐species biofilms as described above. Bacterial suspensions of mono‐ and dual species were added to the plate and incubated for 24, 48 and 72 h at 25 °C. TSB was refreshed every 24 h of incubation. After the incubation time had elapsed, the wells were washed three times in PBS and microscopy was performed. The biofilm architecture was analysed by confocal microscopy using a Leica SP2 (Leica Microsystems, Wetzlar, Germany). Series of images were scanned at 400 Hz using a 63× Leica oil immersion objective (numeric aperture 1.4). The whole well area was inspected to verify the presence of biofilms; then, the most representative location was scanned, providing a stack of horizontal planar images to obtain a three‐dimensional view of the biofilms from the substratum to the top of the biofilms. The argon 488 nm laser was used for excitation of GFP, and the 561 nm laser was used for excitation of the mCherry fluorescent protein. The thickness of the biofilms was measured as the maximal distance between the lowest and highest acquired planar images using confocal microscopy. The thickness was determined as the average value from three locations.

### Scanning electron microscopy

For SEM, dual‐species biofilms were grown on the plastic coverslips as described above. Samples were removed from the dishes at 24, 48 and 72 h, washed three times in PBS and fixed in 3% Millonig phosphate‐buffered glutaraldehyde three times for 10 min (Serva, Heidelberg, Germany) and postfixed in 2% Millonig osmium tetroxide‐buffered solution for 1 h (Serva, Germany). Samples were washed three times for 10 min in Millonig phosphate buffer. The samples were subsequently dehydrated in increasing concentrations of acetone (50, 70, 90 and 100%), every step for 20 min, and dried in hexamethyldisilazane for 3 h in a hood at room temperature (Sigma‐Aldrich, Praha, Czech Republic). Then, the samples were placed on the carbon tabs attached to the aluminium holder and coated with platinum/palladium (Cressington sputter coater 208 HR, UK). The structure and interaction of dual‐species biofilms formed by *S. aureus* and Gram‐negative bacteria were observed under a Hitachi SU 8010 scanning electron microscope (Hitachi High Technologies, Tokyo, Japan) at a magnification of 1500× (at 17 kV, SE+BSE detector, working distance wd 8.4 mm); 6000× (at 15 kV, wd 10.9 mm); 13 000× (at 17 kV, wd 8.4 mm); 30 000× (at 17 kV, wd 13.6).

### Statistical analysis

OD 570 nm values (dependent variable) were analysed by ANOVA (biofilm formers, incubation time as categorical predictors), followed by *post hoc* Bonferroni multiple comparison tests. Logarithmically transformed data of CFU cm^−2^ (dependent variable) were analysed using ANCOVA (with biofilm formers as a categorical predictor and incubation time as a covariate), followed by testing of contrasts between mono‐ and dual‐species biofilms. *P*‐values lower than 0.05 were considered statistically significant. Data analysis was performed using the statistical software statistica 13.0 (Dell, Tulsa, OK, USA).

## Conflict of interest

None declared.
